# Apolipoprotein A1 Gene Polymorphism and Its Association With TNF-Alpha and Interleukin-6 Levels in Uncomplicated Plasmodium Falciparum Malaria in Nigerian Children

**DOI:** 10.12691/ajmsm-13-1-1

**Published:** 2025-02-17

**Authors:** Bose E. Orimadegun, Adedayo O. Faneye, Georgina N. Odaibo, Catherine O. Falade

**Affiliations:** 1 Department of Chemical Pathology, College of Medicine, University of Ibadan, Ibadan, Nigeria; 2 Department of Virology, College of Medicine, University of Ibadan, Ibadan, Nigeria; 3 Department of Pharmacology and Therapeutics, College of Medicine, University of Ibadan, Ibadan, Nigeria

**Keywords:** malaria, apolipoprotein A1, gene polymorphism, tumor necrosis factor-alpha, interleukin-6, inflammation, Nigerian children

## Abstract

Malaria remains a significant health challenge, particularly in sub-Saharan Africa, where it contributes substantially to morbidity and mortality among children. The role of genetic polymorphisms in modulating host responses to malaria has gained attention, with apolipoprotein A1 (APOA1) emerging as a candidate due to its anti-inflammatory and immunomodulatory properties. This study investigates the association between two APOA1 gene polymorphisms (G-75A and C+83T), APOA1 levels, and inflammatory markers (tumor necrosis factor-alpha and interleukin-6) in Nigerian children with uncomplicated Plasmodium falciparum malaria. In this cross-sectional study, 76 children with malaria and 45 healthy controls were recruited. Participants were genotyped for G-75A and C+83T polymorphisms using polymerase chain reaction-restriction fragment length polymorphism. Serum levels of APOA1, interleukin-6, and tumor necrosis factor-alpha were measured using immunoturbidimetric and enzyme-linked immunosorbent assays. Statistical analysis assessed genotype frequencies, inflammatory marker levels, and their associations with parasite burden. The G-75A and C+83T polymorphisms exhibited high frequencies of wild-type alleles (GG and CC, respectively) with no homozygous mutant genotypes observed. APOA1 and inflammatory marker levels differed significantly between children with malaria and controls. APOA1 levels negatively correlated with parasite counts (r = −0.272, p = 0.018), suggesting its role as a negative acute-phase reactant. Tumor necrosis factor-alpha and interleukin-6 levels positively correlated with parasite burden (r = 0.417, p = 0.001 and r = 0.279, p = 0.017, respectively), reflecting their roles in malaria pathogenesis. Gender-specific analysis revealed distinct patterns in biomarker correlations, with males showing stronger inflammatory responses. This study underscores the potential role of APOA1 as a biomarker and therapeutic target in malaria, highlighting the influence of genetic and inflammatory factors on disease severity. These findings provide insights into host-pathogen interactions and may inform personalized strategies for managing malaria in endemic regions.

## 1. Introduction

Malaria remains one of the most formidable public health challenges, particularly in sub-Saharan Africa, where it is a leading cause of morbidity and mortality among children. Caused by the protozoan parasite Plasmodium *falciparum*, malaria is characterized by cyclical fevers, anemia, and, in severe cases, neurological complications and death [1,2]. Different cytokines and acute-phase proteins help the host immune system and the parasite interact in a complex way, initiating a cascade of inflammatory responses [3].

Among the many proteins involved in the immune response to malaria, apolipoprotein A1 (APOA1) has been suspected to play a significant role. APOA1 is the main protein component of high-density lipoprotein (HDL), and it plays a key role in lipid metabolism, reverse cholesterol transport, and controlling inflammatory responses [4]. Beyond its primary functions, APOA1 has antioxidant, anti-inflammatory, and immunomodulatory properties, making it a protein of interest in the study of inflammatory diseases, including malaria [5].

Genetic polymorphisms in the APOA1 gene can lead to variations in the protein expression and function. Two common polymorphisms, G-75A and C+83T, have been linked to changes in blood lipid levels and the risk of heart disease in different populations [6,7]. However, researchers have not extensively studied their role in infectious diseases, particularly malaria. Given the dual role of APOA1 in lipid transport and inflammation, it is plausible that these polymorphisms might influence the inflammatory response in malaria.

Inflammation is a critical component of the immune response to malaria. Key pro-inflammatory cytokines such as tumor necrosis factor-alpha (TNF-α) and interleukin-6 (IL-6) are involved in the pathogenesis of the disease. Elevated levels of TNF-α and IL-6 have been linked to severe malaria and poor clinical outcomes [8,9]. Exploring how variations in the APOA1 gene affect these cytokine levels could help to understand malaria pathogenesis and identify potential biomarkers for disease severity.

This study investigated the association between two common variations in the APOA1 gene (G-75A and C+83T) and serum levels of APOA1, TNF-α, and IL-6 in Nigerian children with Plasmodium *falciparum* infection. By examining these associations, the study seeks to provide insights into the genetic and inflammatory mechanisms underlying malaria and identify potential targets for intervention.

## 2. Materials and Methods

### 2.1. Study Design and Participants

For this cross-sectional study, 76 children with microscopically confirmed malaria parasitaemia and 45 seemingly healthy children of either sex were recruited from an outpatient clinic and a school, respectively. All participants were recruited following informed consent from their parents or guardians. Children were eligible for inclusion if they had microscopically confirmed Plasmodium falciparum parasitaemia, no history of chronic illnesses such as HIV/AIDS, tuberculosis, or sickle cell disease and no prior antimalarial treatment within two weeks before sample collection. Children were excluded if they had mixed Plasmodium infections, were on immunosuppressive therapy, or had received any blood transfusions within the past month and were unwilling or unable to provide consent through their guardians.

### 2.2. Genotyping of APOA1 Polymorphisms

Venous blood samples (5 mL) were collected from each participant using sterile techniques. Blood samples were divided into two aliquots: one for DNA extraction and genotyping, and the other for serum separation. The serum was obtained by centrifuging the blood samples at 3000 rpm for 10 minutes and stored at −80°C until analysis.

Genomic DNA was extracted from whole blood using the QIAamp DNA Blood Mini Kit (QIAGEN, Germany) according to the manufacturer’s instructions. The purity and concentration of the extracted DNA were determined using a NanoDrop spectrophotometer (Thermo Fisher Scientific, USA). The G-75A and C+83T polymorphisms in the APOA1 gene were genotyped using the polymerase chain reaction-restriction fragment length polymorphism (PCR-RFLP) method. Specific primers for the amplification of the target regions were designed as follows:

G-75A: Forward 5′-TCC CAG GAT CCT CTT ACG T-3′, Reverse 5′-AGT GGA GAA CGG TGA GAG TG-3′.

C+83T: Forward 5′-AGG AGG TGA GCC ACA GGA TG-3′, Reverse 5′-GCA GTG CAG GGA CAG GAG AA-3′.

**Figure 1.**
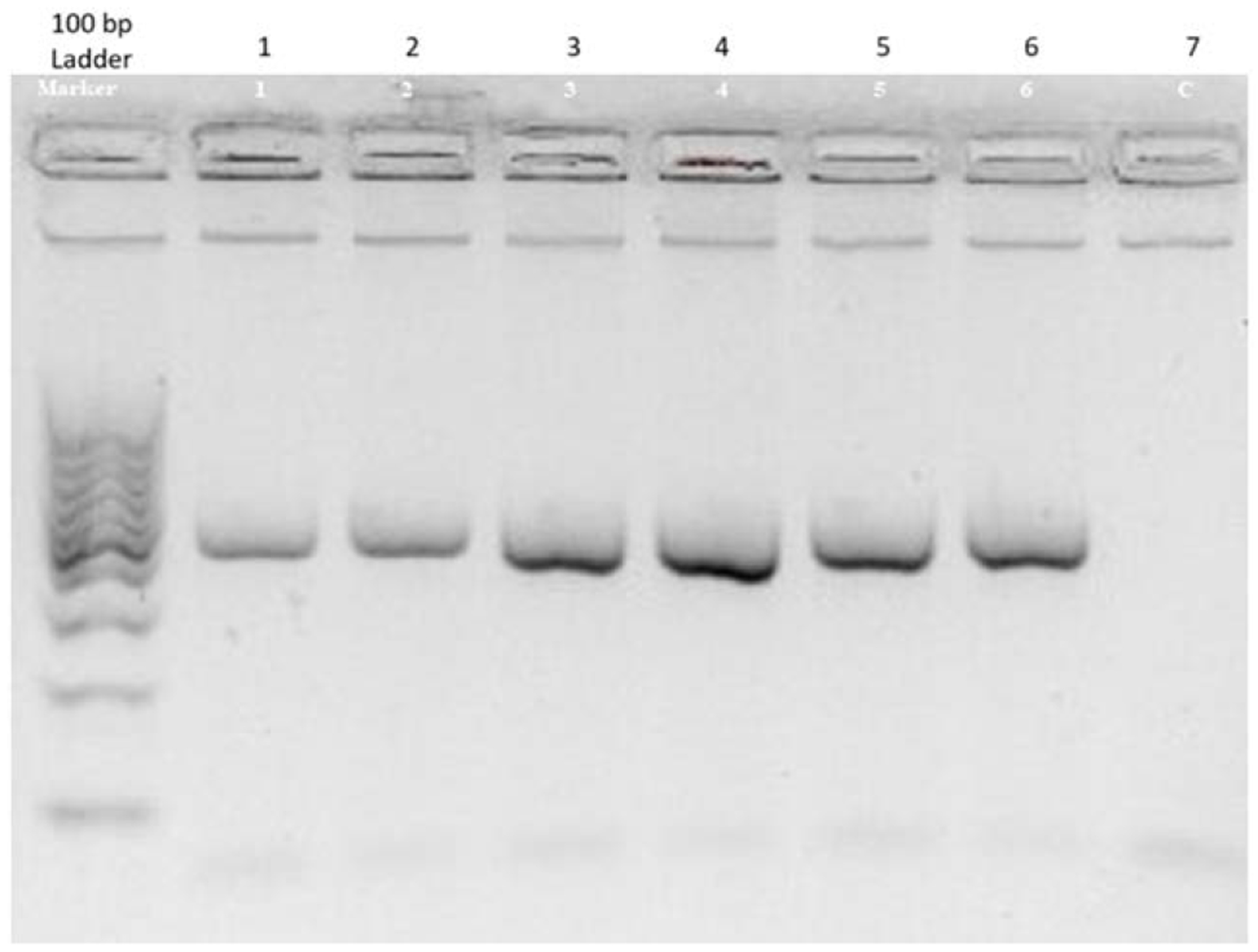
Agarose Gel Electrophoresis of APOA1 PCR Product

PCR amplification was carried out in a total volume of 25 μL containing 2.5 μL of 10X PCR buffer, 2.0 μL of 25 mM MgCl2, 0.5 μL of 10 mM dNTPs, 0.5 μL of each primer (10 μM), 0.25 μL of Taq DNA polymerase (5 U/μL), and 2 μL of genomic DNA (50 ng/μL). The thermal cycling conditions were initial denaturation at 95°C for 5 minutes, followed by 35 cycles of denaturation at 95°C for 30 seconds, annealing at 58°C for 30 seconds, extension at 72°C for 45 seconds, and a final extension at 72°C for 10 minutes. The PCR products were digested with MspI restriction enzyme (New England Biolabs, USA) at 37°C for 4 hours. The digested fragments were analyzed by electrophoresis on a 12% polyacrylamide gel, stained with ethidium bromide, and visualized under UV light. The molecular sizes of the restriction fragments were determined by comparison to a DNA ladder (Figure 1). The expected fragment sizes for each genotype are presented in Table 1. The fragment sizes were compared to a 100 bp DNA ladder to determine the genotypes. The restriction fragment sizes for each genotype were consistent with the expected molecular weights (Table 1).

### 2.2. Measurement of Serum APOA1, TNF-α, and IL-6 Levels

Serum levels of APOA1 were measured using an immunoturbidimetric assay kit (Randox Laboratories, UK) according to the manufacturer’s instructions. The absorbance was read at 340 nm using a microplate reader (Bio-Rad, USA). Serum levels of TNF-α and IL-6 were quantified using enzyme-linked immunosorbent assay (ELISA) kits (R&D Systems, USA). The assays were performed according to the manufacturer’s protocols. Briefly, serum samples were diluted appropriately, and 100 μL of each diluted sample, standard, and control were added to the wells of a pre-coated ELISA plate. After incubation and washing, a biotinylated antibody specific for TNF-α or IL-6 was added, followed by a streptavidin-HRP conjugate. The substrate solution was then added, and the color reaction was stopped with 2N sulfuric acid. The optical density was measured at 450 nm, and the cytokine concentrations were calculated using a standard curve.

### 2.3. Statistical Analysis

Data were analyzed using IBM SPSS Statistics for Windows, version 25 (IBM Corp., Armonk, NY, USA). Descriptive statistics were used to summarize the demographic and clinical characteristics of the study population. Continuous variables were expressed as mean ± standard deviation (SD) or median (interquartile range, IQR), and categorical variables as frequencies and percentages.

The frequency and percentages of categorical variables were calculated. The allelic and genotypic frequencies of the two SNPs, as well as their linkage disequilibrium, were determined. The goodness of fit Chi-square test was used to measure conformity to Hardy Weinberg Equilibrium (HWE). The polymorphism distribution was estimated, and the diplotype frequency of the SNPs was determined. TNF-α, IL-6, and APOA1 levels were also compared using the unpaired t-test or the Kruskal Wallis test as appropriate, while categorical data were compared using the Chi-square tests (with Yates correction wherever required). A p-value of less than 0.05 was considered statistically significant.

### 2.4. Ethical Considerations

Ethical approval (approval number: UI/EC/17/0268) for the study was obtained from the institutional review board of the healthcare facility. The study was conducted following the principles of the Declaration of Helsinki. Written informed consent was obtained from the parents or guardians of all participants before enrolment.

## 3. Results

### 3.1. Characteristics of Participants

The study included 76 children presenting with uncomplicated malaria and 45 serving as healthy controls. The characteristics of the study participants are shown in Table 2. The gender distribution was relatively balanced, with 65 males (53.7%) and 56 females (46.3%) across both groups. Specifically, among those with malaria, 43 (56.6%) were male and 33 (43.4%) were female, whereas in the control group, 22 (48.9%) were male and 23 (51.1%) were female, showing no significant difference in gender distribution between the groups (p = 0.633).

Age analysis revealed a median age of 108.0 months (IQR: 72.0 – 120.0) for the entire cohort. Children with uncomplicated malaria had a slightly lower median age of 96.0 months (IQR: 62.0 – 114.0) compared to the control group, which had a median age of 110.5 months (IQR: 84.0 – 130.0), with this difference reaching statistical significance (p = 0.037).

The mean weight for all participants was 21.9 kg (±6.8). Children with malaria had a mean weight of 20.3 kg (±6.5), while the control group had a higher mean weight of 23.7 kg (±7.0), (p = 0.015). Height-for-age z-scores did not show a significant difference between the groups; the median z-score was −1.28 (IQR: −2.15 to −0.70) for the entire cohort, −1.14 (IQR: −2.16 to −0.73) for the malaria group, and −1.5 (IQR: −2.15 to −0.70) for the control group (p = 0.569).

### 3.2. Inflammatory Markers and Apolipoprotein-A1 (APOA1) Levels

Significant differences were observed in the levels of inflammatory markers and APOA1 between the malaria and control groups. The median IL-6 level for all participants was 14.5 pg/mL (IQR: 2.7 – 1012.5). Children with malaria had markedly elevated IL-6 levels, with a median of 54.2 pg/mL (IQR: 12.84 – 134.36), compared to the control group, which had a median IL-6 level of 3.3 pg/mL (IQR: 2.1 – 14.5), a difference that was significant (p < 0.001).

Similarly, TNF-α levels were significantly higher in the malaria group. The median TNF-α level for all participants was 78.2 pg/mL (IQR: 65.6 – 99.1), with those in the malaria group having a median of 90.8 pg/mL (IQR: 75.4 – 107.5), compared to 48.0 pg/mL (IQR: 35.0 – 78.0) in the control group (p < 0.001). For APOA1, the median level in the cohort was 67.0 mg/mL (IQR: 145.0 – 42.0). In children with malaria, the median APOA1 level was slightly higher at 74.0 mg/mL (IQR: 66.0 – 123.0), whereas the control group had a median level of 69.82 mg/mL (IQR: 58.37 – 78.2), with this difference also being statistically significant (p < 0.001).

### 3.3. Restriction Fragment Analysis of APOA1 Polymorphisms

The analysis of restriction fragment lengths for the G-75A and C+83T polymorphisms in the APOA1 gene revealed distinct patterns for wild homozygote, heterozygote, and mutant homozygote genotypes. For the G-75A polymorphism, the wild homozygote genotype was characterized by fragments of 66 base pairs (bp) and 114 bp. The heterozygote genotype exhibited three fragments: 66 bp, 114 bp, and 180 bp, indicating the presence of both the wild-type and mutant alleles. In contrast, the mutant homozygote genotype displayed a single fragment of 180 bp, representing the complete digestion of the PCR product by the restriction enzyme MspI.

Similarly, the C+83T polymorphism showed distinct fragment sizes based on the genotype. The wild homozygote genotype had fragments of 46 bp and 209 bp. Heterozygotes demonstrated a combination of three fragments: 46 bp, 209 bp, and 225 bp, reflecting the presence of both alleles. The mutant homozygote genotype was identified by a single fragment of 225 bp.

### 3.4. Frequency Distribution of APOA1 Gene Polymorphisms

The frequency distribution of the G-75A and C+83T polymorphisms in the APOA1 gene was analyzed among the study participants, which included 76 children with uncomplicated malaria in Table 3. For the G-75A polymorphism, the genotype frequencies were as follows: 90.8% of the participants were homozygous for the wild-type allele (GG), while 9.2% were heterozygous (GA). No participants were found to be homozygous for the mutant allele (AA). The allelic frequencies for the G-75A polymorphism were 95% for the G allele and 5% for the A allele. There was no significant deviation from Hardy-Weinberg equilibrium for this polymorphism (p = 0.177).

When stratified by gender, the genotype frequencies for males were 95.0% GG and 5.0% GA, with allelic frequencies of 97% for the G allele and 3% for the A allele. In females, the genotype frequencies were 86.1% GG and 13.9% GA, with allelic frequencies of 93% for the G allele and 7% for the A allele. There was a significant deviation from Hardy-Weinberg equilibrium in males (p = 0.026) but not in females (p = 0.200).

For the C+83T polymorphism, 88.2% of the participants were homozygous for the wild-type allele (CC), and 11.8% were heterozygous (CT). No participants were homozygous for the mutant allele (TT). The allelic frequencies for the C+83T polymorphism were 94% for the C allele and 6% for the T allele. There was no significant deviation from Hardy-Weinberg equilibrium for this polymorphism (p = 0.301).

Gender-specific analysis showed that in males, the genotype frequencies were 92.5% CC and 7.5% CT, with allelic frequencies of 96% for the C allele and 4% for the T allele. Among females, the genotype frequencies were 81.1% CC and 18.9% CT, with allelic frequencies of 92% for the C allele and 8% for the T allele. There was no significant deviation from Hardy-Weinberg equilibrium in either males (p = 0.061) or females (p = 0.298).

### 3.5. Correlation of APOA1, Interleukin-6, and TNF-α with Parasite Counts

Table 4 presents the Spearman correlation coefficients between Serum levels of APOA1, IL-6, TNF-α, and parasite counts in children with acute uncomplicated malaria. The analysis was conducted for all participants and separately for males and females.

For the entire cohort, there was a statistically significant negative correlation between APOA1 levels and parasite counts (r = −0.272, p = 0.018). This suggests that higher parasite counts are associated with lower levels of APOA1 in the blood. When analyzed by gender, the negative correlation between APOA1 levels and parasite counts remained for males (r = −0.267), although it did not reach statistical significance (p = 0.169). In females, no significant correlation was observed (r = −0.086, p = 0.696), indicating a potential gender-specific response in APOA1 levels during malaria infection.

IL-6 levels showed a significant positive correlation with parasite counts for the overall cohort (r = 0.279, p = 0.017), indicating that higher parasite loads are associated with increased IL-6 levels. This trend was also observed in males (r = 0.423, p = 0.025), suggesting a stronger inflammatory response in male children. However, the correlation between IL-6 levels and parasite counts in females did not reach statistical significance (r = 0.341, p = 0.111).

TNF-α levels exhibited correlation with parasite counts among the three biomarkers, with a significant positive correlation for the entire cohort (r = 0.417, p = 0.001). This indicates that TNF-α levels rise in conjunction with parasite counts, reflecting the pro-inflammatory role of TNF-α in malaria pathogenesis. Gender-specific analysis revealed no significant correlation for males (r = −0.161, p = 0.412) or females (r = 0.041, p = 0.853), suggesting that the relationship between TNF-α levels and parasite counts might be more complex and influenced by additional factors beyond gender.

### 3.6. Correlation Between Serum TNF-α and APOA1 Levels

**Figure 2.**
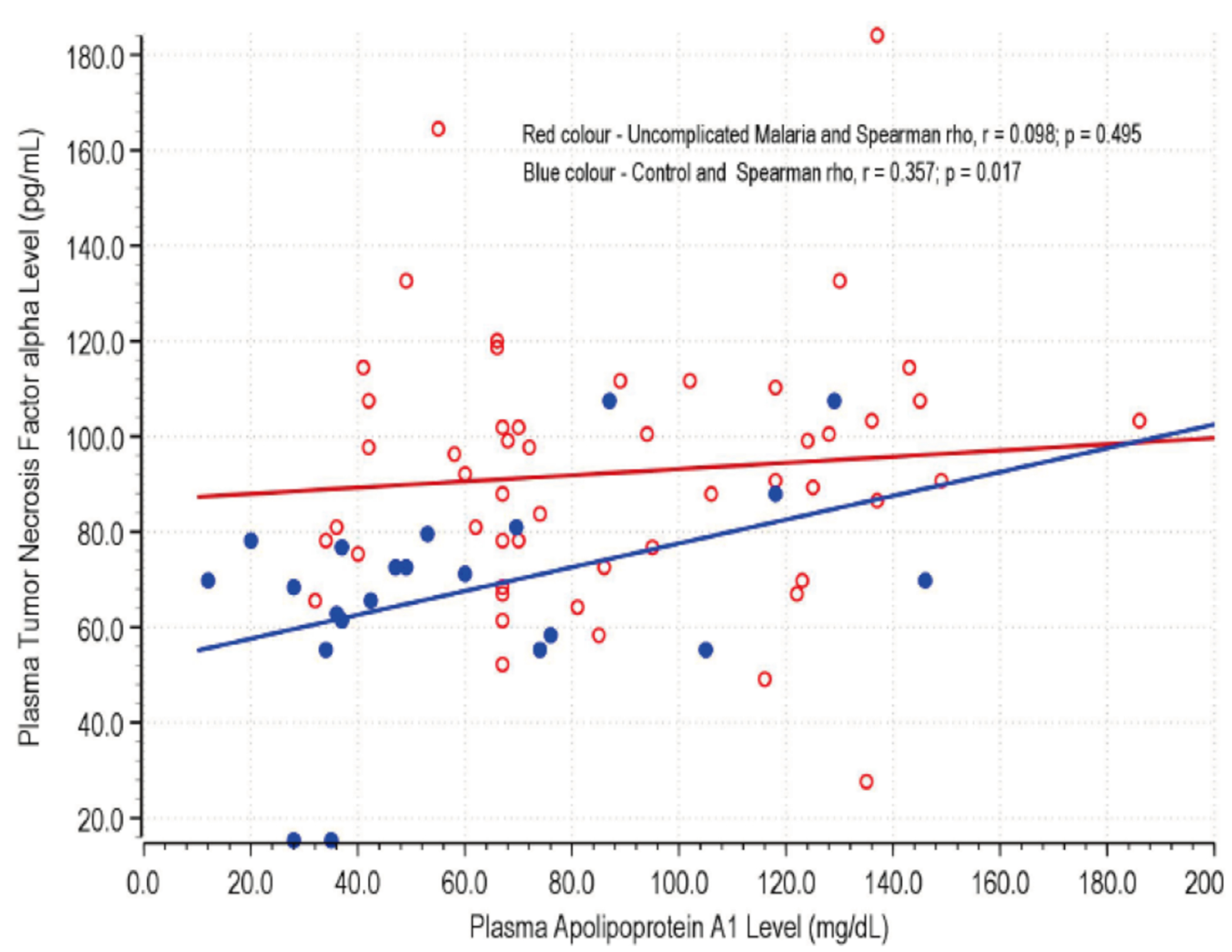
Correlation between Serum TNF-α and APOA1 Levels in Children with Uncomplicated Malaria and Control

Figure 2 illustrates the correlation between Serum TNF-α levels and APOA1 levels in children with uncomplicated malaria compared to control subjects. In the control group, a statistically significant positive correlation was observed between TNF-α and APOA1 levels (Spearman rho r = 0.357, p = 0.017). This indicates that higher levels of TNF-α are associated with higher levels of APOA1 in the control group, reflecting a potential compensatory response in healthy individuals to maintain homeostasis. In contrast, the correlation between TNF-α and APOA1 levels in the uncomplicated malaria group was not statistically significant (Spearman rho r = 0.098, p = 0.495).

### 3.7. Correlation Between Serum APOA1 Levels and IL-6 Levels

**Figure 3.**
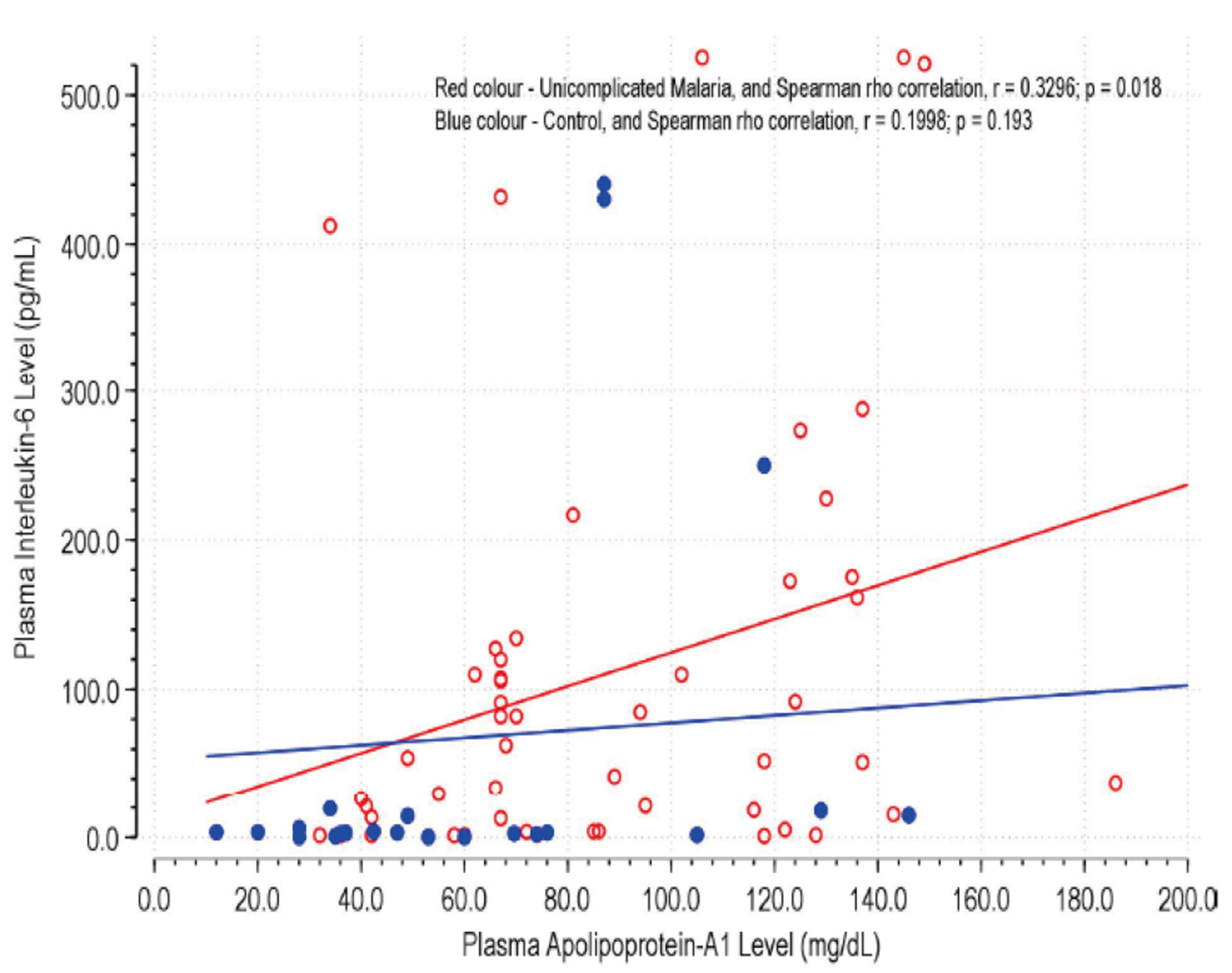
Correlation between Serum Apolipoprotein-A1 Levels and Serum Interleukin-6 Levels in Uncomplicated Malaria and Control Groups

Figure 3 shows the correlation between serum APOA1 levels and IL-6 levels in children with uncomplicated malaria and control for the uncomplicated malaria group, a significant positive correlation was observed (Spearman rho r = 0.3296, p = 0.018), indicating that higher levels of IL-6 are associated with higher levels of APOA1.

In the control group, the correlation between IL-6 and APOA1 levels was not significant (Spearman rho r = 0.1998, p = 0.193). This difference between the two groups highlights the distinct inflammatory profiles in healthy individuals versus those infected with malaria. In healthy controls, IL-6 levels remain relatively low and do not exhibit a strong correlation with APOA1, indicating a stable inflammatory state. However, in malaria-infected children, the positive correlation points to a dynamic response where increased inflammation is accompanied by changes in lipid metabolism and protein expression.

These findings from Figure 2 and Figure 3 collectively demonstrate the complex interplay between inflammatory cytokines and lipid-associated proteins in the context of malaria infection.

## 4. Discussion

### 4.1. Discussion of Findings

In this study, APOA1 gene polymorphisms, serum levels of APOA1, and inflammatory markers (TNF-α and IL-6) were investigated in Nigerian children with uncomplicated malaria. The key findings revealed major differences in the levels of inflammatory markers and APOA1 between children with malaria and healthy controls. The wild-type alleles were more common for the G-75A and C+83T polymorphisms in the APOA1 gene. There was no significant deviation from Hardy-Weinberg equilibrium, except for the G-75A polymorphism in males. Significant correlations were observed between inflammatory markers and parasite counts, with distinct patterns emerging in gender-specific analyses. Altered correlations between inflammatory markers and APOA1 levels were also noted in children with malaria compared to controls.

Baseline characteristics revealed no significant difference in gender distribution between groups, but children with malaria were younger and had lower mean weight compared to controls. These findings align with prior research suggesting that malaria impacts growth and development, with younger children being more susceptible to infections [10]. The absence of significant differences in height-for-age z-scores suggests a less pronounced effect of malaria on long-term growth or the need for longer follow-up to detect such an impact [11].

Children with malaria exhibited higher levels of IL-6 and TNF-α compared to controls, consistent with the inflammatory response characteristic of malaria infection. These cytokines play critical roles in malaria pathogenesis, mediating immune responses and contributing to symptoms such as fever and anemia [12]. Elevated IL-6 levels reflect the acute phase response essential for infection control but also associated with severe complications if dysregulated [13]. High levels of TNF-α and IL-6 in severe malaria cases corroborate previous findings on their role in the malaria pathogenesis [14].

The observed increase in APOA1 levels in malaria patients, despite the inflammatory state, suggests a compensatory mechanism to counteract oxidative and inflammatory stress. Typically, acute inflammation reduces APOA1 levels as HDL components are consumed and modified during immune responses [15]. However, the slight increase in APOA1 levels may indicate its role in mitigating inflammatory damage. This finding aligns with evidence of APOA1’s anti-inflammatory and immunomodulatory properties [16].

Genotype frequencies showed an abundance of wild-type alleles for G-75A and C+83T polymorphisms, with GG and CC being the predominant genotypes. The absence of AA and TT genotypes suggests that mutant alleles are rare in this population. The significant deviation from Hardy-Weinberg equilibrium for the G-75A polymorphism in males may indicate gender-specific genetic factors or selection pressures affecting allele frequencies [17]. Similar patterns of genotype frequencies have been reported in other populations, where wild-type alleles are more prevalent [18,19].

The negative correlation between APOA1 levels and parasite counts suggests that higher parasite burdens are associated with reduced APOA1 levels, supporting its role as a negative acute-phase reactant [20].

The positive correlation between IL-6 levels and parasite counts underscores IL-6 involvement in the inflammatory response to malaria. IL-6 levels rise proportionally with parasite density, reflecting its role in the acute phase response [14]. Stronger correlations observed in males suggest gender-specific differences in inflammatory response, which could influence disease severity and outcomes. TNF-α exhibited the strongest positive correlation with parasite counts, emphasizing its pivotal role in malaria pathogenesis. TNF-α mediates inflammation, fever, and endothelial activation, contributing to malaria complications such as cerebral malaria and severe anemia [21].

The positive correlation between TNF-α and APOA1 levels in the control group suggests a compensatory mechanism to maintain homeostasis. Higher TNF-α levels in healthy individuals are balanced by increased APOA1 levels, which mitigate excessive inflammation [15]. However, this relationship was disrupted in malaria patients, likely due to altered inflammatory and metabolic responses during infection [22].

Strong correlations between IL-6 and APOA1 levels in children with malaria suggest that APOA1 increases as the inflammatory response intensifies, potentially serving as a protective mechanism. This observation aligns with APOA1’s role in modulating inflammation and preventing immune-mediated damage [16]. The absence of this correlation in controls reflects the distinct inflammatory profiles of healthy individuals versus malaria patients, where IL-6 levels remain relatively stable.

### 4.1. Implications of Findings

This study has several important implications. First, the associations between inflammatory markers and parasite counts suggest that IL-6 and TNF-α could serve as biomarkers for malaria severity and therapeutic monitoring. Monitoring these cytokines may help identify children at higher risk of severe outcomes and guide treatment strategies [12]. Second, the correlations between APOA1 and inflammatory markers highlight its potential as a biomarker and therapeutic target in malaria. Given its anti-inflammatory properties, targeting APOA1 pathways could mitigate inflammatory damage in malaria patients [15]. Finally, understanding genotype frequencies of APOA1 polymorphisms provides valuable insights for genetic studies in malaria-endemic regions. These findings may inform personalized medicine approaches and improve malaria management strategies [23].

### 4.3. Strengths and Limitations

A major strength of this study is its comprehensive analysis of APOA1 gene polymorphisms, inflammatory markers, and parasite counts in a well-defined pediatric population. The use of robust techniques, such as ELISA and PCR-RFLP, ensured reliable data. Additionally, gender-specific analyses provided insights into potential hormonal or genetic influences on malaria pathogenesis.

However, the cross-sectional design limits causal inferences between APOA1 polymorphisms, inflammatory biomarkers, and malaria outcomes. Longitudinal studies are needed to explore the dynamic changes in these biomarkers throughout infection and treatment. The relatively small sample size, particularly for genotype analyses, may limit the generalizability of findings.

### 4.4. Future Directions

Future research should focus on longitudinal studies to elucidate causal relationships between APOA1 polymorphisms, inflammatory biomarkers, and malaria outcomes. Larger, more diverse cohorts are necessary to confirm findings and explore the impact of genetic and environmental factors on inflammatory responses. Investigating the functional effects of G-75A and C+83T polymorphisms on APOA1 expression and activity will provide deeper insights into their roles in malaria pathogenesis. Additionally, exploring interactions between APOA1 polymorphisms and other factors, such as diet, co-infections, and prior malaria exposure, will help unravel the multifactorial determinants of inflammation in malaria. Finally, studies on APOA1 mimetics or other anti-inflammatory interventions could pave the way for novel therapeutic strategies to improve malaria outcomes.

### 4.5. Conclusion

This study provides valuable insights into the relationships between APOA1 gene polymorphisms, inflammatory markers, and parasite counts in Nigerian children with uncomplicated malaria. The findings highlight the potential of IL-6, TNF-α, and APOA1 as biomarkers and therapeutic targets in malaria. Further research addressing the limitations of this study will advance our understanding of malaria pathogenesis and inform the development of personalized medical approaches for managing this disease in endemic regions.

## Figures and Tables

**Table 1.** The restriction fragments’ molecular sizes

Polymorphism	Molecular size of restriction fragments (bp)		
	Wild homozygote	Heterozygote	Mutant homozygote
G-75A	66, 114	66, 114, 180	180
C+83T	46, 209	46, 209, 225	225

**Table 2.** Baseline characteristics of the participants

Characteristics	Total (n = 121)	Uncomplicated malaria (n = 76)	Control (n = 45)	p
Male, n (%)	65	43 (56.6)	22 (44.0)	0.633
Age in months	108.0 (72.0, 120.0)	96.0 (62.0, 114.0)	110.5 (84.0, 130.0)	0.037
Weight (kg)	21.9 (±6.8)	20.3 (±6.5)	23.7 (±7.0)	0.015
HAZ-score	−1.28 (−2.15, −0.70)	−1.14 (−2.16, −0.73)	−1.5 (−2.15, −0.70)	0.569
IL-6 (pg/mL)	14.5 (2.7 - 1012.5)	54.2 (12.84–134.36)	3.3 (2.1, 14.5)	<0.001
TNF-α (pg/mL)	78.2 (65.6 - 99.1)	90.8 (75.4–107.5)	48.0 (35.0, 78.0)	<0.001
APOA1 (mg/mL)	67.0 (145.0, 42.0)	74.0 (66.0, 123.0)	69.82 (58.37, 78.2)	<0.001

Values are median and interquartile range (IQR)

**Table 3.** Frequency distribution of genotypes and alleles of the G-75A and C+83T Polymorphisms

	Genotype frequency				Allelic frequency		

	n	GG	GA	p	G	A	
G-75A							χ^2^
All participants	76	69 (90.8)	7 (9.2)		0.95	0.05	0.177
Male	40	38 (95.0)	2 (5.0)	0.346	0.97	0.03	0.026
Female	36	31 (86.1)	5 (13.9)		0.93	0.07	0.200
C+83T	n	CC	CT		C	T	χ^2^
All participants	76	67 (88.2)	9 (11.8)	0.379	0.94	0.06	0.301
Male	40	37 (92.5)	3 (7.5)		0.96	0.04	0.061
Female	37	30 (81.1)	6 (8.9)		0.92	0.08	0.298

* Yates corrected Chi-square test for deviation from Hardy-Weinberg equilibrium

**Table 4.** Spearman’s correlations of APOA1, Interleukin-6, TNF-α with parasite counts in children with acute uncomplicated malaria

	All participants		Male		Female	

	r	P	r	p	r	p
APOA1	−0.272	0.018	−0.267	0.169	−0.086	0.696
Inteleukin-6	0.279	0.017	0.423	0.025	0.341	0.111
TNF-α	0.417	0.001	−0.161	0.412	0.041	0.853
